# Real-World Effectiveness and Safety of Liuwei Dihuang Pill for Menopausal Syndrome: Protocol for a Prospective, Observational, Multicenter Cohort Study

**DOI:** 10.2196/84803

**Published:** 2026-03-25

**Authors:** Shuoshuo Wei, Hongyan Zhang, Mengmeng Wang, Yuhui Sun, Tao Song, Weifang Zheng, Yunmei Ye, Liran Ma, Yunshuang Yang, Tong Wei, Lan Cheng, Chunquan Sun, Xingting Ding, Shichu Zhao, Qingqiao Song, Lianxin Wang

**Affiliations:** 1 Institute of Basic Research in Clinical Medicine, China Academy of Chinese Medical Sciences Beijing China; 2 First Affiliated Hospital of Harbin Medical University Heilongjiang China; 3 Lanxi Hospital of Traditional Chinese Medicine Zhejiang China; 4 Beijing Gulou Hospital of Traditional Chinese Medicine Beijing China; 5 Beijing Longfu Hospital Beijing China; 6 Dongcheng District Hepingli Community Health Service Center Beijing China; 7 Beijing Puren Hospital Beijing China; 8 Tsinghua University Yuquan Hospital (Tsinghua University Hospital of Integrative Traditional Chinese and Western Medicine) Beijing China; 9 Dongcheng District Chaoyangmen Community Health Service Center Beijing China; 10 Guang’anmen Hospital, China Academy of Chinese Medical Sciences Beijing China

**Keywords:** Liuwei Dihuang pill, real world, menopausal syndrome, protocol, effectiveness, safety

## Abstract

**Background:**

Menopausal syndrome (MPS) results from declining ovarian function and estrogen fluctuations during the menopausal transition, typically presenting with vasomotor, psychological, and metabolic symptoms that impair quality of life. Current treatments, including hormone replacement therapy and nonhormonal medications, are limited by safety concerns and adverse effects. The Liuwei Dihuang (LWDH) pill, a classical traditional Chinese medicine formula, is widely used for kidney-yin deficiency and menopausal symptoms such as hot flashes, insomnia, depression, and anxiety. Preliminary studies suggest favorable efficacy and safety; however, robust real-world evidence, particularly in combination with conventional Western therapy, remains insufficient.

**Objective:**

This study aims to evaluate the real-world effectiveness and safety of LWDH in combination with conventional Western therapy for MPS.

**Methods:**

This prospective, multicenter, real-world, observational cohort study will be conducted at 8 centers in China from March 9, 2025, to December 2026. A total of 1000 patients with MPS in a real-time clinical setting will be allocated to either the exposure group (conventional therapy plus LWDH) or the nonexposure group (conventional therapy alone). The primary outcome is the change in the modified Kupperman Index. The secondary outcomes encompass changes in the Menopausal Quality of Life Scale, serum sex hormone levels, traditional Chinese medicine syndrome scores, and metabolic parameters. Metabolomics analyses will be conducted to explore potential mechanisms. Safety will be assessed by monitoring adverse events and clinically significant abnormalities in vital signs or laboratory parameters. Assessments will be conducted at baseline and at 2 and 4 weeks, with a subsequent 2-week follow-up. Propensity score–based inverse probability of treatment weighting will be applied to balance baseline covariates, and weighted regression models will be used for comparative analyses.

**Results:**

Participant recruitment and enrollment began in March 2025. It is expected that by December 2026, a total of 1000 participants will be recruited. At present, patient recruitment is ongoing, and preliminary organization and analysis of medical record data are underway. Blood sample collection for metabolomics analysis is also in progress. Final data collection is expected to be completed in March 2027. We expect to generate evidence to support clinical decision-making, with results anticipated to be published in esteemed, peer-reviewed health research journals by December 2027.

**Conclusions:**

This study will assess the clinical effectiveness, safety, and potential mechanisms of LWDH in the treatment of MPS in real-world settings, providing a scientific basis for its use in clinical practice.

**Trial Registration:**

ClinicalTrials.gov NCT06874738; https://clinicaltrials.gov/study/NCT06874738

**International Registered Report Identifier (IRRID):**

DERR1-10.2196/84803

## Introduction

Menopausal syndrome (MPS) arises from declining ovarian function and estrogen fluctuations during the perimenopausal transition, primarily manifesting as vasomotor symptoms (hot flashes and night sweats), psychological disturbances (anxiety and depression), and metabolic disorders (dysglycemia and dyslipidemia) [1,2]. It is estimated that by 2030, more than 1.2 billion women worldwide will be in the menopausal or postmenopausal stage, with up to 85% experiencing menopausal symptoms to varying degrees [3,4]. Hormonal alterations, particularly involving the hypothalamic-pituitary-ovarian axis, may furthermore increase the risk of glycolipid metabolic disorders, cardiovascular diseases, osteoporosis, and neurocognitive disorders, underscoring the substantial public health burden of MPS [5-7].

Current management primarily includes hormone therapy and nonhormonal pharmacological agents. Hormone therapy mitigates symptoms resulting from diminished estrogen levels by providing estrogen and progesterone; however, its prolonged use correlates with an elevated risk of breast cancer and adverse cardiovascular events [8,9]. Antidepressants, gabapentin, and clonidine are nonhormonal medications that can ameliorate symptoms such as hot flashes and mood fluctuations, but they may also cause side effects such as nausea, insomnia, and dry mouth [10,11]. In addition, owing to individual variability among patients, symptoms may recur after the discontinuation of these treatments. Therefore, safer and more sustainable therapeutic strategies are still required.

Traditional Chinese medicine (TCM) has a long history and rich experience in treating MPS, with its advantages lying in the emphasis on holistic management, which offers it unique benefits and broad prospects for application in this domain. In ancient China, MPS was viewed as a category of “depression syndrome, insomnia, and fatigue” based on its characteristic symptoms and signs. The principal causes of this condition were thought to be deficiencies in the liver and kidneys [12]. Treatment methods included acupuncture, *tuina* (Chinese therapeutic massage), moxibustion, dietary therapy, and herbal medicine, all aimed at nourishing and tonifying these organs [13,14]. According to literature survey, herbal medicines can successfully alleviate symptoms and enhance quality of life, exhibit few side effects, and show high patient compliance, rendering it suitable for long-term use [15,16]. It was found that many formulas used in the treatment of MPS due to kidney-yin deficiency are based on the foundational recipe of Liuwei Dihuang (LWDH), which shows significant therapeutic effects [17,18].

LWDH originates from the medical text “Xiao Er Yao Zheng Zhi Jue,” written by the renowned physician Qian Yi during the Song Dynasty. This formula is composed of 6 traditional Chinese herbs: *Rehmannia glutinosa* (Shudihuang), *Cornus officinalis* (Shanzhuyu), *Dioscorea opposita* (Shanyao), Poria (Fuling), *Alisma orientale* (Zexie), and Moutan Cortex (Mudanpi). Each ingredient contributes to its therapeutic effects, targeting nourishing yin and tonifying the kidneys [18]. Modern pharmacological studies indicate that LWDH exhibits a range of effects, including antioxidant, anti-inflammatory, and antitumor properties, along with antihyperglycemic and lipid-lowering effects [19-21]. Although prior clinical trials have suggested potential effectiveness of LWDH for MPS [22,23], these studies were conducted under highly controlled conditions with stringent inclusion and exclusion criteria, limited treatment flexibility, and relatively homogeneous patient populations, which may constrain external validity and generalizability to routine clinical practice. In real-world settings, treatment decisions for MPS—especially regarding TCM—are influenced by patient preferences, symptom burden, prior treatment history, and shared decision-making between clinicians and patients. Imposing random allocation in this context may reduce patient willingness to participate and interfere with authentic prescribing behaviors, thereby limiting the study’s ability to capture real-world treatment patterns. Accordingly, this multicenter real-world study will enroll 1000 patients with MPS to evaluate the effectiveness and safety of LWDH in routine clinical settings, aiming to provide pragmatic evidence for its integration into MPS management.

## Methods

### Research Design

This is a multicenter, prospective, real-world, nonrandomized controlled study (registered at ClinicalTrials.gov; NCT06874738) designed to evaluate the effectiveness and safety of LWDH in patients with MPS associated with kidney-yin deficiency.

A total of 1000 patients with MPS associated with kidney-yin deficiency will be recruited from 8 centers across China and divided into 2 cohorts: exposure group (patients receiving conventional Western medical therapy plus LWDH) and nonexposure group (patients receiving conventional Western medical therapy alone). Physicians will determine whether to prescribe LWDH based on clinical assessment, guideline recommendations, and patient preference. Assessments will be conducted at baseline and at 2, 4, and 6 weeks after enrollment, with the aim of systematically evaluating the real-world effectiveness of LWDH and further exploring its potential mechanisms of action.

**Figure 1:**
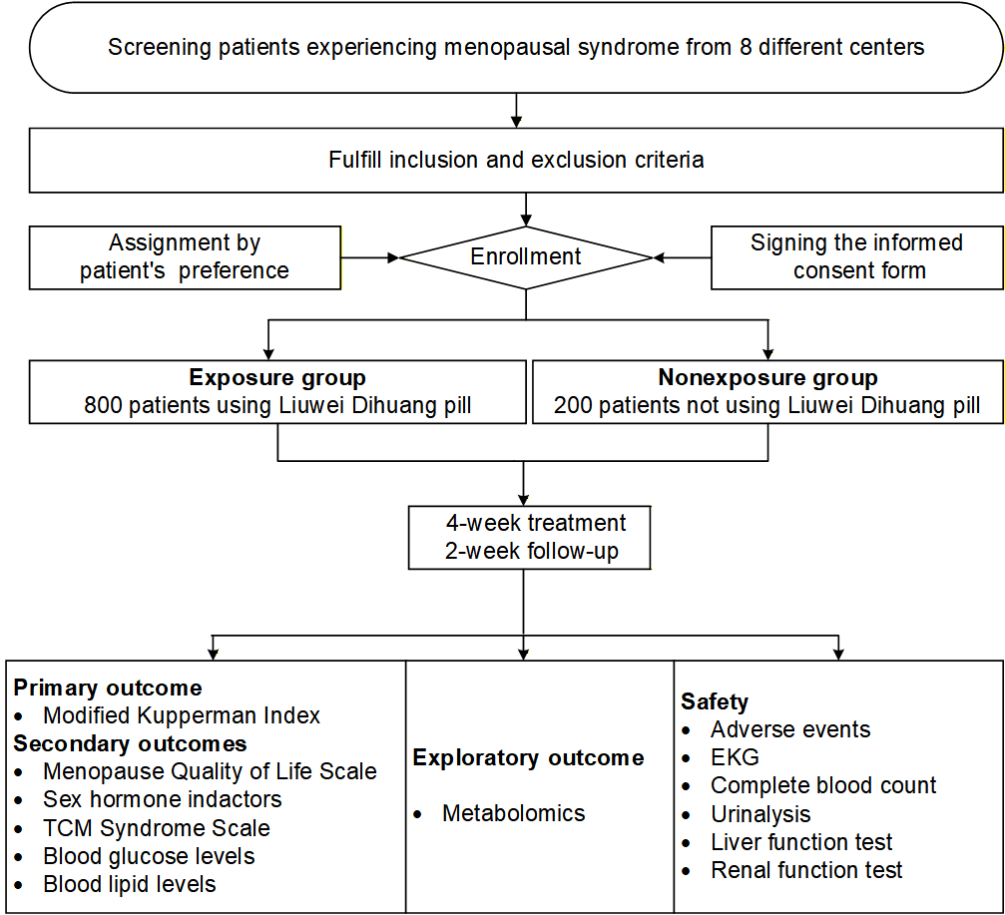
Flow diagram of the study. EKG: electrocardiography; TCM: traditional Chinese medicine.

**Figure 2:**
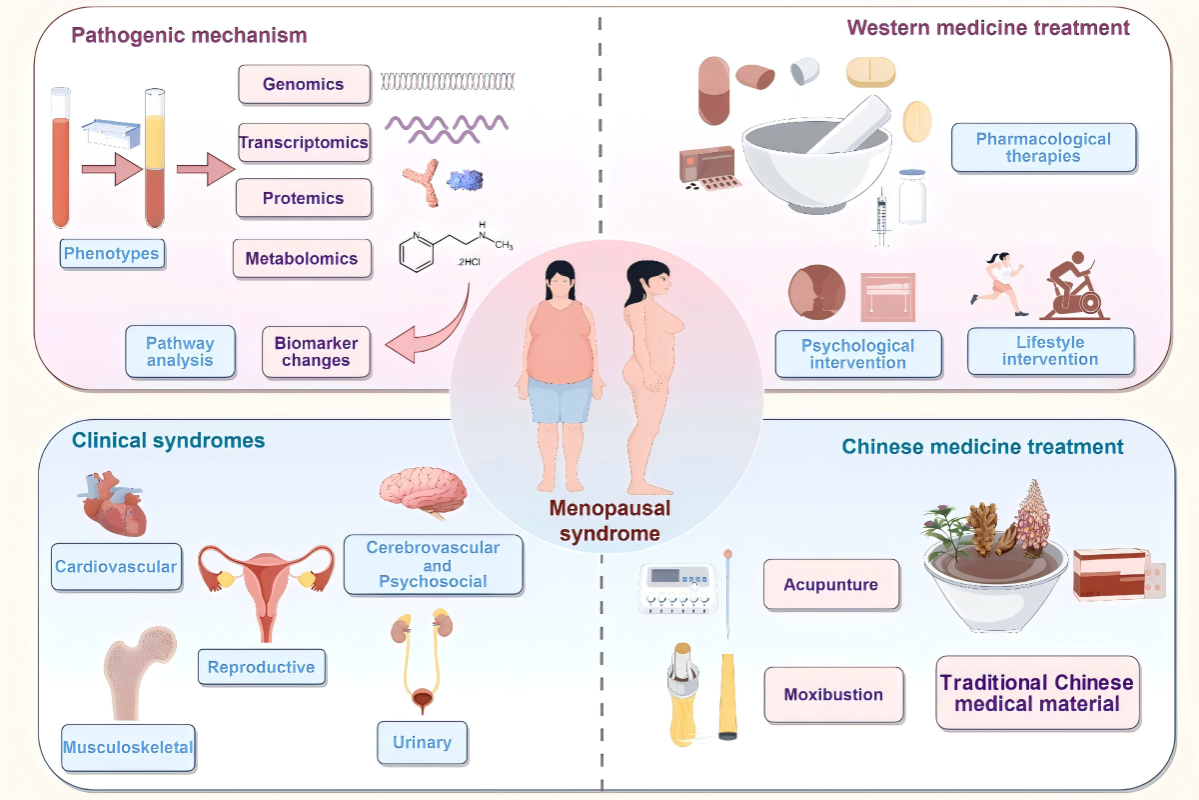
Summary of the protocol.

The study protocol adheres to the SPIRIT (Standard Protocol Items: Recommendations for Interventional Trials) statement [24]. The completed SPIRIT checklist is provided as Multimedia Appendix 1. The study flowchart and key elements of the protocol are presented in Figures 1 and 2.

### Allocation Strategy and Blinding of Outcome Assessment

As this is a real-world observational study, treatment allocation is determined by routine clinical decision-making and patient preference; therefore, randomization and participant blinding are not applicable.

To minimize potential analytical bias, statisticians and outcome assessors involved in data analysis will remain blinded to group identification during statistical evaluation.

### Sample Size Calculation

The primary objective of this trial is to evaluate the therapeutic effectiveness of LWDH in patients with MPS. Sample size estimation was based on the assumption that the exposure group would demonstrate superior improvement in the modified Kupperman Index compared with the nonexposure group. On the basis of prior clinical data [25], we assumed that the mean difference in the change of the modified Kupperman Index between the 2 groups (δ) would be 0.6, with a common SD (σ) of 2.

The sample size calculation was performed using a 2-sample independent *t* test, with a 2-sided significance level (α) of .05 and a statistical power (1−β) of 0.90. Participants in the exposure and nonexposure groups will be included in a 4:1 ratio (K) [26]. The required sample size per group was calculated using the following formula for a superiority trial:

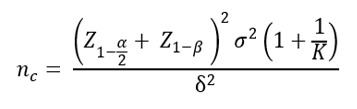



On the basis of these assumptions, the estimated sample size was 640 participants in the exposure group and 160 participants in the nonexposure group. Considering an anticipated dropout rate of 20%, the final sample size was adjusted accordingly. Therefore, the planned sample sizes were set at 800 participants in the exposure group and 200 participants in the nonexposure group, yielding a total sample size of 1000 participants.

### Study Participants

This study focuses on MPS in patients diagnosed with a TCM pattern of kidney-yin deficiency. These patients receive treatment in the outpatient departments of 8 institutions: Beijing Longfu Hospital, Beijing Gulou Traditional Chinese Medicine Hospital, Lanxi Traditional Chinese Medicine Hospital, The First Affiliated Hospital of Harbin Medical University, Beijing Dongcheng District Hepingli Community Health Service Center, Beijing Dongcheng District Chaoyangmen Community Health Service Center, Beijing Puren Hospital, and Tsinghua University Yuquan Hospital (Tsinghua University Hospital of Integrative Traditional Chinese and Western Medicine).

### Inclusion Criteria

Participants are deemed to be in perimenopause according to Stages of Reproductive Aging Workshop classification [27] and meet all the following criteria [28-30]: (1) women aged 45 to 55 years (inclusive), (2) diagnosis of MPS according to Western medical diagnostic criteria, (3) presence of menstrual disturbances or amenorrhea lasting more than 3 months, (4) diagnosis of kidney-yin deficiency syndrome according to TCM syndrome differentiation criteria, (5) a modified Kupperman Index score of 6 or higher, (6) serum follicle-stimulating hormone level higher than 10 IU/L, and (7) voluntary provision of written informed consent.

### Exclusion Criteria

Participants meeting any of the following criteria will be excluded from the trial: (1) those with known allergies to the ingredients of LWDH or contraindications to Chinese herbal preparations; (2) women who are pregnant, lactating, or intending to conceive; (3) patients with severe primary disorders of the liver, kidney, and hematopoietic system; and (4) patients with comorbidities that significantly impact assessment, including severe cognitive impairment and aphasia.

### Treatments

Eligible individuals who provide informed consent will be assigned to either the exposure group (conventional Western therapy plus LWDH) or the nonexposure group (conventional Western therapy alone) based on their preferences and whether they are taking LWDH in real clinical practice. All participants will receive guideline-based conventional Western medical therapy tailored to their clinical condition [13,31,32]. In addition, patients in the exposure group will receive LWDH at a dose of 8 pills (3 g) orally, 3 times daily for 4 weeks.

### Concomitant Medications

During the entire monitoring phase, the use of combination therapy and other treatments is permitted, whereas the use of traditional Chinese herbal formulas with yin-nourishing and kidney-tonifying effects is strictly prohibited.

### Outcomes

#### Primary Outcome

The primary outcome is as follows: the modified Kupperman Index will be used to quantify the overall severity of MPS. This index comprises 13 items covering vasomotor, somatic, and psychological symptoms, with weighted scores ranging from 0 to 63; higher scores indicate greater symptom severity.

#### Secondary Outcomes

The secondary outcomes are as follows:

- The Menopause Quality of Life scale will be assessed as a key patient-reported secondary outcome. The scale includes 29 items across 4 domains (ie, vasomotor, psychosocial, physical, and sexual), with lower scores indicating better quality of life.
- The 6 sex hormone indicators will be used to characterize endocrine levels during the perimenopausal period, including follicle-stimulating hormone, luteinizing hormone, estradiol, progesterone, testosterone, and prolactin.
- TCM syndrome scores for kidney-yin deficiency will be evaluated using a standardized TCM Syndrome Scale. This scale evaluates 3 dimensions: physical sensations, psychological feelings, and urogenital symptoms, with higher scores indicating more severe symptoms.
- Blood glucose levels will be assessed using fasting blood glucose, 2-hour postprandial blood glucose, and glycated albumin to provide insights into the state of glucose metabolism.
- Blood lipid levels will be assessed using total cholesterol, triglycerides, high-density lipoprotein cholesterol, and low-density lipoprotein cholesterol to help evaluate the status of lipid metabolism.

#### Exploratory Outcomes

Metabolomics analysis will be conducted as an exploratory secondary outcome, focusing on the preliminary exploration of molecular signals potentially related to treatment response and metabolic changes.

All efficacy outcomes will be assessed at baseline and at 4 weeks after treatment initiation.

#### Safety Outcomes

Safety assessments will include monitoring adverse events, vital signs (blood pressure and heart rate), routine laboratory tests (hematology, urinalysis, and hepatic and renal function), and electrocardiography. Adverse events will be recorded throughout the study and followed until resolution or stabilization.

### Data Collection and Study Procedures

The data collection and follow-up schedule is presented in Table 1.

**Table 1:** Time schedule of participant enrollment and follow-up.

			Screening and enrollment		Treatment phase				Follow-up
			Baseline		2 weeks +7 to −7 days		4 weeks +7 to −7 days		6 weeks +7 to −7 days
**Enrollment**									
	Confirm eligibility	✓							
	Informed consent	✓							
	Demographics	✓							
	Medical history-treatment history-medication history	✓							
**Treatment**									
	LWDH^a^			✓		✓			
**Assessments**									
	Vital signs	✓		✓		✓			
	Hematological examination	✓		✓		✓			
	Questionnaire surveys	✓		✓		✓			
	EKG^b^	✓		✓		✓			
	Prescription and distribution of LWDH	✓		✓					
	Evaluation of medication compliance			✓		✓			
	Combined medication	✓		✓		✓			
	Adverse events			✓		✓		✓	
	Changes in medical history, treatment, or medication			✓		✓		✓	
	Conclusion of completion of the case							✓	

^a^LWDH: Liuwei Dihuang.

^b^EKG: electrocardiography.

#### Screening and Enrollment Phase

Participants will be screened to determine their eligibility based on inclusion and exclusion criteria. Before administering the medication, data will be collected on general demographic information (eg, age, gender, ethnicity, occupation, height, and weight), vital signs (eg, temperature, blood pressure, heart rate, and respiratory rate), medical history, treatment history, and medication history. Patients enrolled in the study will undergo hematological tests (sex hormone test, complete blood count, urinalysis, blood lipid test, blood glucose test, liver function test, and renal function test), electrocardiography, and questionnaire assessments (modified Kupperman Index, Menopausal Quality of Life Scale, TCM Syndrome Scale). The collected information will be recorded in the screening and enrollment section of the case report forms (CRFs).

#### Treatment Phase

Follow-up visits will occur at 2 weeks (±7 days) and 4 weeks (±7 days) after enrollment. At each visit, vital signs, laboratory parameters, questionnaire assessments, adverse events, medical history, medication history, and treatment history since enrollment will be recorded. Furthermore, information regarding the prescription and distribution of LWDH will also be collected at screening and enrollment and the first follow-up visit. When LWDH is newly prescribed, its use will be assessed at the second follow-up visit and the final follow-up visit. Relevant information will be written in the treatment section of CRFs.

#### Follow-Up Visit

A telephone follow-up will be conducted at 6 weeks (+7 to −7 days) after enrollment to monitor adverse events and changes in clinical status or medication use. Study completion status will be documented accordingly.

### Serum Sample Collection and Preparation

All blood tests will be conducted after an 8-hour fasting period and a minimum of 30 minutes of rest before collecting venous blood. For menopausal participants, blood samples will be taken before treatment and on the last day of treatment, while for menstruating participants, blood samples will be collected on the second day of their menstrual period. Investigators will collect morning fasting elbow venous blood samples (2-3 mL) from the 2 cohorts at 2 time points (before and after treatment). After 40 minutes at room temperature, the samples will be centrifuged at 3000 rpm at 4 °C for 10 minutes. The upper serum will be aliquoted into four 1.5 mL Eppendorf tubes (500 μL per tube), labeled, and stored at –80 °C for metabolomics analysis.

### Data Entry and Quality Control

#### Data Management

The following procedures will be implemented to ensure data management:

- Study data will be initially recorded in CRFs and subsequently entered into a secure electronic data capture (EDC [33]) system. All data entry personnel will receive standardized training before study initiation. Double data entry with cross-verification will be implemented to ensure accuracy.
- The EDC system incorporates automated logic checks to identify missing values, inconsistencies, and outliers. Key variables, including reproductive hormone measurements, will undergo additional verification. Source documents (eg, informed consent forms, laboratory reports, and electrocardiography) will be retained for quality review.
- Data cleaning will include consistency checks, missing data assessment, outlier detection, and duplicate record screening. The final database will be locked after joint review and confirmation by the principal investigator and the statistician.

#### Quality Control Measures

All study procedures will be conducted in accordance with standardized operating procedures. Quality assurance measures will be implemented throughout the study to ensure data integrity and protocol compliance.

#### Investigator Training

Investigators at each center will receive protocol-specific training before study initiation. Ongoing updates related to study procedures will be communicated as necessary throughout the trial.

### Participant Adherence and Safety Monitoring

#### Enhancing Participant Adherence

To enhance adherence, participants will receive standardized instructions regarding medication use and study procedures at enrollment. Reminder strategies (eg, telephone calls or text messages) will be implemented to promote follow-up attendance and treatment compliance. Medication use and symptom changes will be recorded at each visit.

#### Adverse Event Monitoring

Adverse events will be collected through scheduled assessments and spontaneous reports. Investigators will evaluate their severity and potential relationship to study treatment and manage them according to routine clinical practice. Serious adverse events will be reported to the ethics committees in accordance with regulatory requirements.

### Statistical Analysis

#### Statistical Software and Significance Level

Statistical analyses will be performed using SPSS Statistics (version 26.0; IBM Corp) and R software (version 4.5.1; R Foundation for Statistical Computing). All statistical tests will be 2-sided, and a significance level (α) of .05 will be applied.

#### Descriptive Analysis

Descriptive analyses will be conducted to summarize the demographic and baseline characteristics of the participants. Continuous variables will be expressed as means (SDs) or medians (IQRs), as appropriate, while categorical variables will be presented as frequencies and percentages.

#### Primary Analysis and Control of Confounding

Given the nonrandomized, real-world design, the primary comparative analysis will be conducted using inverse probability of treatment weighting (IPTW) based on propensity scores to control for potential confounding and selection bias. Propensity scores will be estimated using a multivariable logistic regression model including a priori–selected baseline covariates, such as age, BMI, duration of MPS, comorbidities, and concomitant hormonal therapy and other pharmacological therapies (including the type of hormonal therapy, if applicable). The distribution and overlap of propensity scores between groups will be examined to assess the positivity assumption, and covariate balance after weighting will be assessed using standardized mean differences and other balance diagnostics. Univariable and multivariable analyses will be conducted to explore associations between baseline covariates and outcomes and to inform model specification; however, the primary comparative analyses will be based on IPTW-adjusted models. Covariate balance before and after weighting will be evaluated using standardized mean differences. A standardized mean difference of less than 0.1 will be considered indicative of adequate balance. A covariate balance table summarizing baseline characteristics before and after IPTW will be presented in the final study report to demonstrate the effectiveness of the weighting procedure. In addition, graphical diagnostics will be used to visually assess balance across covariates.

#### Outcome-Specific Analytical Methods

Analytical methods will be selected according to the type of outcome variables. Continuous outcomes, including scale scores, sex hormone levels, and metabolic indicators, will be analyzed using weighted linear regression models or analysis of covariance, with adjustment for baseline values when applicable. Categorical outcomes will be analyzed using weighted chi-square tests or weighted logistic regression models. Secondary unweighted analyses will be performed for descriptive and exploratory purposes. Normality of continuous variables will be assessed using the Shapiro-Wilk test. For normally distributed variables, between-group comparisons will be conducted using independent-samples 2-tailed *t* tests and within-group comparisons will be performed using paired-samples 2-tailed *t* tests. For nonnormally distributed variables, the Mann-Whitney *U* test will be applied.

#### Situations and Handling of Missing Values

Data missingness is described as variables explicitly stated as missing or unobtainable, unreported variables (ie, blank observations), reported data that are ambiguous, or data that are inconsistent or beyond acceptable ranges.

Missing data will be handled using multiple imputation under the assumption of missing at random for the primary analysis. As a sensitivity analysis, the last observation carried forward method will also be applied, and results obtained using different imputation methods will be compared to assess robustness.

#### Sensitivity Analyses

To further evaluate the robustness of the primary findings, sensitivity analyses will be conducted using alternative propensity score approaches, including propensity score matching and stratification. Additional sensitivity analyses will be performed by excluding participants receiving concomitant hormonal therapy and by restricting analyses to patients with full treatment adherence.

#### Consideration of Center Effects

Given the multicenter prospective design, potential center effects will be considered in all adjusted analyses. Study center will be included as a covariate or treated as a stratification factor, as appropriate, to account for intercenter heterogeneity. Stratified analyses across study centers may be conducted when the sample size permits.

#### Reporting of Results

Effect estimates will be reported with corresponding 95% CIs and *P* values.

### Serum Metabolomics Analysis

Serum samples stored at −80 °C will be thawed and sorted on ice according to their respective groups. Subsequently, 30 μL of serum will be transferred into an Eppendorf tube, and 120 μL of prechilled 80% methanol containing internal standard I will be added for extraction. The samples will then be centrifuged at 4 °C at 18,000×*g* for 30 minutes, and the supernatant will be collected. EDC and 3-nitrophenylhydrazine will be added to the supernatant for derivatization for 60 minutes. The reaction will be terminated by adding an equal volume of ice-cold 50% methanol, followed by centrifugation at 4 °C at 4000×*g* for 30 minutes. The resulting supernatant will be collected, internal standard II will be added, and the mixture will be prepared for injection. Analysis will be performed on an ultra-high performance liquid chromatography coupled to a quadrupole or time-of-flight high-resolution mass spectrometer (positive and negative ion modes). Raw metabolomics data will be preprocessed with MassLynx 4.1 (Waters Corporation) and imported into SIMCA-*P* 14.1 (MKS Umetrics) for Orthogonal Partial Least Squares Discriminant Analysis. Differential metabolites will be identified against the Human Metabolite Database (University of Alberta). Finally, pathway enrichment of differential metabolites will be performed using KEGG (Kanehisa Laboratories) via MetaboAnalyst 5.0 (McGill University).

### Ethical Considerations

This study has been approved by the Ethics Committee of Beijing Longfu Hospital (LFYYLL-2025-02) and will be conducted in accordance with the Declaration of Helsinki and Good Clinical Practice guidelines (Multimedia Appendix 2). Written informed consent will be obtained from all participants before enrollment (Multimedia Appendix 3). Participants may withdraw from the study at any time without penalty. No financial or material compensation will be provided for participation. Venous blood sampling may cause temporary discomfort, bruising, or mild dizziness. The study will not provide participants with any medications. To safeguard data security, direct identifiers are removed at collection and replaced with coded IDs; the coding key is stored separately, with access limited to authorized staff. An ethics-approved, comprehensive data storage and management plan has been implemented to minimize any risk of breach.

## Results

The study protocol (version 1.0) was finalized in February 2025. Participant recruitment began in March 2025 across 8 participating centers and is expected to be completed by December 2026. Final data collection, including all follow-up assessments, is anticipated to be completed in March 2027. As of February 2026, a total of 500 participants have been enrolled, with 420 (84%) in the LWDH exposure group and 80 (16%) in the nonexposure group. Among them, 230 (46%) participants have completed follow-up assessments (n=200, 87% in the exposure group and n=30, 13% in the nonexposure group). In addition, pretreatment and posttreatment blood samples have been collected from 20 (4%) participants for metabolomics analysis (n=15, 75% in the exposure group and n=5, 25% in the nonexposure group). We expect the future results of this study to be published in esteemed, peer-reviewed health research journals by December 2027.

## Discussion

### Expected Findings

We hypothesize that patients receiving LWDH in routine clinical practice may experience greater improvement in menopausal symptoms, hormone indices, and quality of life compared with those receiving standard Western medical care alone. In addition, exploratory serum metabolomics analysis is expected to provide preliminary insights into metabolic pathways potentially involved in symptom improvement.

### Comparison to Prior Work

Previous studies have suggested that LWDH may alleviate menopausal symptoms through modulation of endocrine function and metabolic homeostasis [34]; however, most available evidence is derived from randomized controlled trials conducted under controlled conditions. In contrast, this study adopts a prospective, real-world design with preference-based treatment allocation, aiming to complement existing evidence by assessing treatment effectiveness under routine clinical conditions. By integrating clinical outcomes with exploratory metabolomics analysis, this study seeks to bridge clinical effectiveness research and mechanistic hypothesis generation.

### Strengths and Limitations

The strengths of this study include its prospective real-world design; comprehensive collection of clinical and laboratory outcomes; and the application of advanced statistical methods, such as propensity score–based IPTW, to mitigate confounding associated with nonrandomized group allocation.

Several limitations should also be acknowledged. First, despite statistical adjustment, residual confounding due to unmeasured factors cannot be fully excluded, and causal inference should therefore be interpreted with caution. Second, key outcomes rely on self-reported scales, which may introduce subjective reporting bias. Third, the relatively short follow-up period focuses on short-term effectiveness and safety and does not allow evaluation of long-term outcomes or delayed adverse effects.

### Future Directions

Future studies with longer follow-up durations, larger sample sizes, and more rigorous designs are warranted to confirm the sustainability of treatment effects and to further investigate the mechanistic pathways suggested by the exploratory metabolomics findings. These results may inform the design of subsequent randomized trials or longitudinal cohort studies integrating clinical outcomes with molecular profiling.

### Dissemination Plan

The findings of this study will be disseminated through peer-reviewed publications and academic conferences focusing on integrative medicine, menopausal health, and real-world evidence research. The results may also inform clinical decision-making and future guideline development for the management of MPS.

### Conclusions

This prospective, multicenter, real-world cohort study will provide the first large-scale clinical evidence regarding the effectiveness and safety of LWDH in women with MPS due to kidney-yin deficiency. By integrating clinical outcomes with exploratory serum metabolomics analysis, the study aims to generate real-world comparative effectiveness evidence and preliminary hypotheses regarding potential metabolic pathways associated with treatment response. The findings will offer robust evidence to support clinical practice, promote precision medicine, and facilitate the development of integrative strategies for improving women’s health during the menopausal transitions.

## Data Availability

Data sharing is not applicable to this paper as no datasets were generated or analyzed during this study.

## Appendix

Multimedia Appendix 1 SPIRIT checklist.

Multimedia Appendix 2 Ethics statement.

Multimedia Appendix 3 Informed consent form.

## Abbreviations

CRF: case report form
EDC: electronic data capture
IPTW: inverse probability of treatment weighting
LWDH: Liuwei Dihuang
MPS: menopausal syndrome
SPIRIT: Standard Protocol Items: Recommendations for Interventional Trials
TCM: traditional Chinese medicine
