# Correction: Real-World Effectiveness and Safety of Liuwei Dihuang Pill for Menopausal Syndrome: Protocol for a Prospective, Observational, Multicenter Cohort Study

**DOI:** 10.2196/84867

**Published:** 2026-04-21

**Authors:** Shuoshuo Wei, Hongyan Zhang, Mengmeng Wang, Yuhui Sun, Tao Song, Weifang Zheng, Yunmei Ye, Liran Ma, Yunshuang Yang, Tong Wei, Lan Cheng, Chunquan Sun, Xingting Ding, Shichu Zhao, Qingqiao Song, Lianxin Wang

**Affiliations:** 1 Institute of Basic Research in Clinical Medicine, China Academy of Chinese Medical Sciences Beijing China; 2 First Affiliated Hospital of Harbin Medical University Heilongjiang China; 3 Lanxi Hospital of Traditional Chinese Medicine Zhejiang China; 4 Beijing Gulou Hospital of Traditional Chinese Medicine Beijing China; 5 Beijing Longfu Hospital Beijing China; 6 Hepingli Community Health Service Center Dongcheng District, Beijing China; 7 Beijing Puren Hospital Beijing China; 8 Tsinghua University Yuquan Hospital (Tsinghua University Hospital of Integrative Traditional Chinese and Western Medicine) Beijing China; 9 Chaoyangmen Community Health Service Center Dongcheng District, Beijing China; 10 Guang’anmen Hospital, China Academy of Chinese Medical Sciences Beijing China

In “Real-World Effectiveness and Safety of Liuwei Dihuang Pill for Menopausal Syndrome: Protocol for a Prospective, Observational, Multicenter Cohort Study” [1], the authors made several corrections.

The following changes were made to the author affiliations:

First, the affiliation for author Y Ye has been updated. Y Ye is affiliated with institution 3. Institution 4 in the original manuscript has been removed.

Second, affiliation 6 has been corrected from the following:

Hepingli Community Health Service Center, Beijing, China

Affiliation 6 now reads:

Hepingli Community Health Service Center, Dongcheng District, Beijing, China

Third, affiliation 7 has been corrected from the following:

Beijing Purun Hospital, Beijing, Beijing, China

Affiliation 7 now reads:

Beijing Puren Hospital, Beijing, China

Fourth, affiliation 8 has been corrected from the following:

Tsinghua University Yuquan Hospital (Tsinghua University Hospital of Integrative Traditional Chinese and Western Medicine), Beijing, Beijing, China

Affiliation 8 now reads:

Tsinghua University Yuquan Hospital (Tsinghua University Hospital of Integrative Traditional Chinese and Western Medicine), Beijing, China

Fifth, affiliation 9 has been corrected from the following:

Chaoyangmen Community Health Service Center, Beijing, China

Affiliation 9 now reads:

Chaoyangmen Community Health Service Center, Dongcheng District, Beijing, China

Finally, affiliation 10 has been corrected from the following:

Guang’anmen Hospital Jinan Hospital, China Academy of Chinese Medical Sciences, Beijing, China

Affiliation 10 now reads:

Guang’anmen Hospital, China Academy of Chinese Medical Sciences, Beijing, China

The corrected author affiliations will appear as follows:

Shuoshuo Wei^1^, MM; Hongyan Zhang^1^, MMSc; Mengmeng Wang^1^, MMSc; Yuhui Sun^2^, MD; Tao Song^2^, MD; Weifang Zheng^3^, MBBS; Yunmei Ye^3^, MBBS; Liran Ma^4^, MM; Yunshuang Yang^5^, PhD; Tong Wei^6^, MM; Lan Cheng^7^, MM; Chunquan Sun^8^, MD; Xingting Ding^9^, MM; Shichu Zhao^7*^, PhD; Qingqiao Song^10*^, MD; Lianxin Wang^1*^, PhD

^1^Institute of Basic Research in Clinical Medicine, China Academy of Chinese Medical Sciences, Beijing, China

^2^First Affiliated Hospital of Harbin Medical University, Heilongjiang, China

^3^Lanxi Hospital of Traditional Chinese Medicine, Zhejiang, China

^4^Beijing Gulou Hospital of Traditional Chinese Medicine, Beijing, China

^5^Beijing Longfu Hospital, Beijing, China

^6^Hepingli Community Health Service Center, Dongcheng District, Beijing, China

^7^ Beijing Puren Hospital, Beijing, China

^8^Tsinghua University Yuquan Hospital (Tsinghua University Hospital of Integrative Traditional Chinese and Western Medicine), Beijing, China

^9^Chaoyangmen Community Health Service Center, Dongcheng District, Beijing, China

^10^Guang’anmen Hospital, China Academy of Chinese Medical Sciences, Beijing, China

^*^these authors contributed equally

In the Study Participants and Acknowledgments section, the hospital name was corrected from the following:

Beijing Purun Hospital

The hospital name has been corrected to the following:

Beijing Puren Hospital

These corrections do not affect the scientific conclusions of the article.

The correction will appear in the online version of the paper on the JMIR Publications website, together with the publication of this correction notice. Because this was made after submission to PubMed, PubMed Central, and other full-text repositories, the corrected article has also been resubmitted to those repositories.
