# Supplementary material for: Real-World Effectiveness and Safety of Liuwei Dihuang Pill for Menopausal Syndrome: Protocol for a Prospective, Observational, Multicenter Cohort Study
Source: JMIR Res Protoc. 2026 Mar 25;15:e84803. doi: 10.2196/84803 (PMC13062740; doi:10.2196/84803)
Supplement: Multimedia Appendix 2 [file resprot_v15i1e84803_app2.docx]

****Ethics Statement****

**This study was approved by the Ethics Committee of Beijing Longfu Hospital (approval number: LFYYLL-2025-02; approval date: February 12, 2025). The study will be conducted in accordance with the Declaration of Helsinki, the International Council for Harmonisation Good Clinical Practice (ICH-GCP) guidelines, and relevant Chinese regulations governing human biomedical research ethics.**

**All participants will provide written informed consent prior to enrollment. Participants will be fully informed about the study objectives, procedures, potential benefits and risks, data confidentiality measures, and their right to withdraw at any time without penalty.**

**Data protection and confidentiality: All personal information will be de-identified and coded to ensure participant privacy. Data will be stored securely in encrypted databases with restricted access limited to authorized research personnel only.**

**Ongoing ethical oversight: This study is subject to annual continuing review by the Ethics Committee of Beijing Longfu Hospital, with progress reports submitted every 12 months as required, or more frequently if needed. Any protocol amendments or serious adverse events will be reported to the ethics committee promptly.**
