# Supplementary material for: Real-World Effectiveness and Safety of Liuwei Dihuang Pill for Menopausal Syndrome: Protocol for a Prospective, Observational, Multicenter Cohort Study
Source: JMIR Res Protoc. 2026 Mar 25;15:e84803. doi: 10.2196/84803 (PMC13062740; doi:10.2196/84803)
Supplement: Multimedia Appendix 3 [file resprot_v15i1e84803_app3.doc]

**Informed Consent Form • Informed Consent Page**

Dear Sir/Madam,

We would like to invite you to participate in a clinical study titled "Real-World Clinical Study on the Treatment of Menopausal Syndrome with Liuwei Dihuang Pill."

Before you decide whether or not to participate in this study, please read the following information carefully. It will help you understand the purpose of the study, the procedures and duration involved, as well as the potential benefits, risks, and discomforts that may arise from participating. If you wish, you can discuss this with your family or friends, or ask your doctor for further clarification to assist in making an informed decision.

**1.Study Introduction**

Menopausal syndrome is a series of symptoms related to autonomic dysfunction caused by a decrease in sex hormones before and after menopause. Common symptoms include menstrual irregularities, hot flashes, night sweats, sleep disturbances, and emotional disorders. The core cause is the gradual decline in ovarian function, which includes both natural and surgical menopause. Due to the irreversible low levels of estrogen, post-menopausal aging can last for at least 30-40 years. The post-menopausal aging phase occupies about one-third or more of a woman's lifespan, during which the incidence of complications significantly increases. Every year, approximately 25 million women worldwide enter menopause, and in China, 10 million women reach menopause annually. The World Health Organization estimates that by 2030, the global population of menopausal women will exceed 1.2 billion, with over 210 million menopausal women in China, accounting for about 1/7 of the total population, highlighting the importance of addressing this issue in public health.

Western medical treatments for menopausal syndrome mainly involve psychological therapy and hormone replacement therapy (HRT). However, hormone supplementation often leads to diseases in other organs, such as breast cancer and cervical cancer, making it a limited approach. Traditional Chinese Medicine (TCM) has been used for thousands of years to treat menopausal syndrome, referred to as "symptoms before and after the cessation of menstruation" in TCM. It falls under categories such as "Yuhe Disease" and "Zangzao." The fundamental pathogenesis of the disease involves kidney deficiency and the decline of "Tian Gui" (essence of life), leading to an imbalance of kidney yin and yang, which is a key factor in causing the various symptoms before and after menopause. Kidney yin and yang imbalances often affect other organs, primarily the heart, liver, and spleen. If kidney yin is insufficient and cannot nourish the heart, this leads to an overactive heart fire. The liver and kidney are both related in terms of yin, and a deficiency of kidney yin results in insufficient essence to generate blood, leading to liver-kidney yin deficiency, which causes liver yang to rise. Furthermore, the kidney and spleen mutually support each other, with spleen yang relying on kidney yang for warmth. When kidney yang is weak, fire cannot warm the earth, leading to kidney-spleen yang deficiency, which may cause dampness, phlegm, blood stasis, and qi stagnation, resulting in complex pathological mechanisms and diverse clinical symptoms.

Liuwei Dihuang Pill is first recorded in the Xiaor Yao Zhi Jue (Manual of Pediatric Drug Prescriptions) and is a classic formula for tonifying the kidneys. It is listed in the first batch of the Ancient Classic Formulas Directory by the National Administration of Traditional Chinese Medicine. The formula consists of six herbs: Shu Di Huang (Rehmannia), Shan Yao (Chinese yam), Mu Dan Pi (Moutan Cortex), Shan Zhi Yu (Cornus fruit), Fu Ling (Poria), and Ze Xie (Alisma). It has the effect of nourishing yin and tonifying the kidneys, and is used for symptoms such as dizziness, tinnitus, sore lower back and knees, hot flashes, night sweats, seminal emissions, and thirst due to kidney yin deficiency.

Shu Di Huang nourishes yin and tonifies the kidneys, while Fu Ling helps to expel dampness and strengthen the spleen. Shan Yao nourishes the spleen yin, and Shan Zhi Yu tonifies the liver and kidneys. Mu Dan Pi clears and dispels fire from the heart, which balances the warming and astringent properties of Shan Zhi Yu. Ze Xie promotes urination and clears kidney dampness, reducing the overly rich nature of Shu Di Huang. The formula also has immune-enhancing, blood lipid-lowering, blood pressure-reducing, anti-fatigue, anti-aging, low-temperature resistance, and oxygen deficiency resistance properties. It improves kidney function and autonomic nervous system function, promotes metabolism, and increases estrogen levels in menopausal women. It stimulates the feedback regulation of the hypothalamus-pituitary-ovarian axis, improves endocrine function, balances the body’s internal environment, and alleviates clinical symptoms.

Chinese medicine, with its distinct approach based on syndrome differentiation and treatment, offers a unique and effective treatment for menopausal syndrome, and clinical observations and research have confirmed its definite efficacy and good safety profile.

**2. Introduction of the Study Medication**

[Study Medication Name] Liuwei Dihuang Pill

[Ingredients] Shu Di Huang (Rehmannia), Shan Yao (Chinese yam), Mu Dan Pi (Moutan Cortex), Shan Zhi Yu (Cornus fruit), Fu Ling (Poria), Ze Xie (Alisma)

[Indications] Nourishes yin and tonifies the kidneys. Used for kidney yin deficiency, sore lower back and knees, night sweats, and seminal emissions.

[Specifications] As labeled in the medication instructions.

[Dosage and Administration] Oral administration, according to the dosage indicated in the medication instructions.

[Storage] Seal tightly.

[Appearance] Brownish-black to black honey pill; sweet and slightly sour taste.

[Contraindications] Contraindicated for those allergic to the ingredients of this medication.

[Adverse Reactions] Monitoring data indicates that Liuwei Dihuang preparations may cause diarrhea, abdominal pain, bloating, nausea, vomiting, gastrointestinal discomfort, loss of appetite, constipation, itching, rash, headache, palpitations, and allergic reactions.

[Precautions]

1. Avoid spicy and hard-to-digest foods.
2. Do not take cold medications during treatment.
3. If symptoms such as loss of appetite, gastric discomfort, diarrhea, or abdominal pain occur during medication, seek medical attention.
4. If symptoms do not improve after 2-4 weeks of treatment, consult a doctor.
5. Take the medication as instructed. Children, pregnant women, and breastfeeding women should take it under the guidance of a doctor.
6. Contraindicated for those allergic to this product, use cautiously for those with allergic constitution.
7. Do not use if the product has changed in appearance.
8. Children must use the medication under adult supervision.
9. Keep the medication out of reach of children.
10. If taking other medications, consult a doctor or pharmacist before using this product.
11. If adverse reactions occur during medication, discontinue use. If symptoms persist, consult a doctor.
12. Those with severe conditions such as hypertension, heart disease, liver disease, diabetes, or kidney disease should take it under the guidance of a doctor.

This study is part of the National Key R&D Program on Traditional Chinese Medicine Modernization, "Establishment and Demonstration of Key Technologies for Clinical Efficacy and Safety Evaluation of Chinese Patent Medicines Based on Systems Biology" (No. 2022YFC3502004), funded by the project “Clinical Validation of Chinese Patent Medicine Efficacy and Safety and Discovery of Biomarkers.” The study has been reviewed by the Ethics Committee of Beijing Longfu Hospital and complies with relevant national regulations and ethical principles in the Helsinki Declaration to protect the rights of research participants.

**3. Inclusion and Exclusion Criteria**

3.1Inclusion Criteria:

(1) Aged 45-55 years (inclusive), female;

(2)Meets the Western medical diagnostic criteria for menopausal syndrome;

(3)Meets the Traditional Chinese Medicine diagnostic criteria for kidney yin deficiency syndrome;

(4)Informed consent and voluntary signing of the consent form.

# 3.2 Exclusion Criteria:

(1)Known allergy to any ingredient in Liuwei Dihuang Pill or contraindications for traditional Chinese medicine formulations;

(2) Pregnant or breastfeeding women, or women planning pregnancy;

(3)Patients with serious primary diseases such as liver, kidney, or hematopoietic system disorders;

(4) Patients with comorbidities that significantly affect the assessment, such as severe cognitive impairment or aphasia.

# **4. What Will You Need to Do If You Participate in the Study?**

If you meet the inclusion criteria and agree to participate, the study will proceed as follows:

Pre-medication (Day 0):(1)Formal enrollment: Eligible participants will be enrolled.(2)Blood sample collection and testing, completion of efficacy and safety assessments, and review of qualified study subjects for formal enrollment.(3)Complete the CRF (Case Report Form).(4)Start taking Liuwei Dihuang Pill according to the doctor’s prescription and your own preferences (this study does not provide the medication). (5)Set the next follow-up appointment.

Post-medication (4 weeks):(1)Collect blood samples again for testing.(2)Complete the CRF again.

Additional matters that require your cooperation:Timely follow-up visits and reporting of symptom improvements.Blood sample collection at specified time points.

# **5. Benefits of Participating in the Study**

By participating in this clinical study, your condition may improve.You will receive free tests related to this study, including:Six hormone tests (FSH, LH, estradiol, progesterone, testosterone, prolactin); Routine blood tests (hemoglobin, red blood cells, platelets, white blood cells, neutrophils, lymphocytes); Urinalysis (urine white blood cells, red blood cells, protein); Liver and kidney function tests (ALT, AST, creatinine, blood urea nitrogen); Blood lipid tests (total cholesterol, triglycerides, HDL-C, LDL-C); Blood glucose tests (HbA1c, fasting blood glucose, postprandial blood glucose at 2 hours); Electrocardiogram (ECG);Consultation and feedback on omics test results. Your participation will also contribute to the real-world clinical study of Liuwei Dihuang Pill in treating menopausal syndrome, which is of social significance for the treatment of this disease and for other patients with similar conditions.

**6. Risks of Participating in the Study**

This study will involve drawing about 2.5 ml of blood before and after medication. The blood collection process may have minor risks, including brief pain, local bruising, and mild dizziness in some cases. If you experience any discomfort during the study, please immediately inform your study doctor, who will assess and provide medical treatment for any discomfort.

# **7. Costs, Compensation, and Reimbursement**

Participating in this study will provide you with free laboratory tests, consultation services, and omics test feedback. Treatment and tests for other concurrent diseases will not be covered by this study.

**8. Is Personal Information Confidential?**

Your information will be recorded in the research medical record / case report form. All research data in the original medical records (including personal information, test reports, etc.) will be kept strictly confidential within the legal limits. Your name will not appear on the CRF; only your name initials and the number assigned to you during the study will be used. In any related research summaries, publications, or public documents, only your initials and number may be mentioned.

When necessary, regulatory agencies, ethics committees, or the funding organization may review your study data as required. However, they will not use your data for any other purposes or disclose it to other organizations without permission.

**9. How to Obtain More Information?**

You may ask any questions regarding this study at any time.

Your doctor will provide you with his/her contact number to answer any questions.

If any important new information arises during the study that may affect your willingness to continue, your doctor will inform you promptly.

**10. Voluntary Participation and Withdrawal from the Study**

Participation in this study is entirely voluntary. You may refuse to participate or withdraw at any time during the study without any impact on your medical treatment or rights. If you choose to withdraw, you will not be subject to any discrimination or retaliation.

Your doctor or the researcher may also discontinue your participation for your best interests. If you withdraw from the study for any reason, the doctor may consult with you regarding your use of the study medication. You may also be asked to undergo laboratory tests and physical examinations. You have the right to refuse, and this will not result in any discrimination or retaliation.

**11. What Should You Do Now?**

The decision to participate in this study is entirely up to you. You can discuss it with your family or friends before making your decision.

Please feel free to ask your doctor any questions to ensure that you fully understand the study before deciding whether to participate.

**12. Ethics Committee**

If you have any questions or need to consult someone other than the researcher, please contact the Ethics Committee of Beijing Longfu Hospital.

Ethics Committee Office: Beijing Longfu Hospital, Science and Education Department

Phone: 87947345

Thank you for reading the above information. If you decide to participate in this study, please inform your doctor, and they will arrange everything for you.

Please keep this document for your records.

**Informed Consent Form • Signature Page**

Study Title:Real-World Clinical Study on the Treatment of Menopausal Syndrome with Liuwei Dihuang Pill

Project Source:National Key Research and Development Program of China“Modernization of Traditional Chinese Medicine” Project (No. 2022YFC3502004)

### **Statement of Consent**

I have read the above information describing this study and have had the opportunity to discuss the study and ask questions with the physician.
 All of my questions have been answered to my satisfaction.

1. I understand the potential risks and benefits of participating in this study. I understand that my participation is voluntary, and I confirm that I have had sufficient time to consider my decision. I understand that:

(2) I may ask my physician for additional information at any time.

(3) I may withdraw from this study at any time without discrimination or retaliation, and my medical care, rights, and interests will not be affected.

(4) I also understand that if I withdraw from the study prematurely, particularly due to reasons related to the study medication, informing my physician of any changes in my condition and completing the relevant physical and laboratory examinations will be beneficial to both myself and the study as a whole.

If I require any other medications for the treatment of my illness, I will consult my physician in advance or inform my physician truthfully afterward.

I agree that representatives of the drug regulatory authorities, the ethics committee, or the funding agency may review my study-related records as required.

I will receive a signed and dated copy of this informed consent form.

Finally, I voluntarily agree to participate in this study and will make every effort to comply with the physician’s instructions.

**Participant’s Signature:** __________________________

**Date:** ______ Year ______ Month ______ Day

**Participant’s Contact Number:** ______________________

I confirm that I have explained the details of this study to the participant, including their rights and the potential benefits and risks, and have provided the participant with a signed copy of the informed consent form.

**Investigator’s Signature:** __________________________

**Date:** ______ Year ______ Month ______ Day

**Investigator’s Contact Number:** _____________________
